# The Effect of Leucocytosis, Gender Difference, and Ultrasound in the Diagnosis of Acute Cholecystitis in the Elderly Population

**DOI:** 10.1155/2019/6428340

**Published:** 2019-04-02

**Authors:** Arda Demirkan, Ayça Koca Tanrıverdi, Arda Çetinkaya, Onur Polat, Müge Günalp

**Affiliations:** ^1^Ankara University, School of Medicine, General Surgery Department, Samanpazarı, Ankara, Turkey; ^2^Ankara University, School of Medicine, Emergency Department, Samanpazarı, Ankara, Turkey

## Abstract

**Introduction:**

Acute cholecystitis is one of the most common reasons of acute abdominal pain for older patients to present to the emergency department (ED). Presentation may differ from that of the younger patient and is often complicated by coexistent disease due to elderliness. In this study, we aimed to evaluate the clinical presentation of acute cholecystitis, with special focus on comparision between elderly and young patients.

**Materials and Methods:**

This study included 318 patients who were admitted to the emergency department with right upper quadrant pain during a period of determined 8 months. After retrospective data collection, patients were groupped in accordance with their age, <65 and ≥65 years. Those who had ultrasonographic signs such as wall thickening and fluid collection were diagnosed as acute cholecystitis.

**Results:**

The young group (Group I) consisted of 225 patients, 132 females and 93 males. In Group I, 39 patients were diagnosed as acute cholecystitis of whom 27 were females and 15 were males. The elderly group (Group II) consisted of 93 patients 48 females and 45 males. In Group II, 36 patients were diagnosed as acute cholecystitis of whom 15 were females and 21 were males. Regarding the diagnosis of acute cholecystitis, the female to male ratio is 2.25 in Group I and 0.71 in Group II (p=0.016). The average white blood cells counts of patients with acute cholecystitis in Group I and in Group II were 9907x10^9^/L(±4.437) and 17083x10^9^/L(±7485), respectively (p<0,001).

**Conclusions:**

Acute cholecystitis is a common diagnosis in elderly patients with right upper quadrant pain. It is more frequent in female in the early ages, but the gender difference tends to change with age. Elderly patients demonstrate a higher level of white blood cells when compared to young patients in acute cholecystitis. Clinicians must maintain a degree of awareness in the evaluation of geriatric patients with right upper quadrant abdominal pain.

## 1. Introduction

Approximately 40% of all patients admitting to the emergency department are older than 65 years of age and because of an increasingly aging population, this proportion is steadily increasing [1, 2]. Age-related physiologic changes affect nearly every organ system and influence the presentation of diseases. A specific emphasis should be placed on characterizing the differences in the clinical presentation and diagnostic accuracy between the younger and more elderly patients [3, 4]. Right upper quadrant pain is a common complaint and this type of pain can be caused by a wide variety of conditions, but one of the foremost disease processes in the mind of the evaluating physician is likely to be acute cholecystitis. The typical presentation of a patient with acute cholecystitis is pain in the right upper quadrant, usually accompanied by fever, nausea, and vomiting. The presentation of an older patient with acute cholecystitis may be very different and a significant number of these patients do not have classic symptoms of cholecystitis because of coexisting disease or diminished ability to localize acurately pain [5–8]. Changes in fever may not be correlated with the severity of the infection [9, 10]. An incomplete or ambiguous history as well as atypical and subtle physical findings complicates the diagnostic process in the elderly. The clinical picture is even further complicated by preexisting conditions and medications. This study was designed to assess the differences between elderly patients and their younger counterparts who presented with a complaint of right upper quadrant abdominal pain and who were diagnosed to have acute cholecystitis.

## 2. Materials and Methods

The study was conducted at the Emergency Department of the Ankara University School of Medicine. Database and files were retrospectively reviewed with local ethics committee approval. Our study population was selected from patients who were admitted with a complaint of right upper quadrant (RUQ) pain between June 2007 and January 2008. In this time period, all the patients were diagnosed by the same physician and abdominal ultrasounds were performed by the same radiologist team in our emergency department (ED). Patients undergoing abdominal ultrasonography for the evaluation of right upper quadrant pain were considered eligible. The medical records of these patients were reviewed retrospectively and information regarding fever, laboratory values including white blood cells (WBC), aspartate aminotransferase (AST), alanine aminotransferase (ALT), alkaline phosphatase (ALP), and total bilirubin, and abdominal ultrasonography findings were obtained. The records of all patients presenting to the ED with RUQ pain were reviewed. The diagnosis of acute cholecystitis was made by means of a clinical picture, physical examination, laboratory tests, and abdominal ultrasonography. Diagnostically ultrasonography is the modality of choice for acute cholecystitis [11].

The ultrasonographic criteria used to diagnose acute cholecystitis included “the finding of gallstones with significant wall thickening over 5 mm, pericholecystic fluid, impacted stone, or a combination of these parameters” [12].

In the absence of gallstones, gallbladder wall thickening with localized gallbladder tenderness and pericholecystic fluid was considered indicative of acalculous cholecystitis. Choledocholithiasis, biliary pancreatitis, acalculous cholecystitis, gallbladder cancer, gallbladder polyps, and other extra and intraabdominal pathologies causing right upper quadrant abdominal pain were exclusion criteria. A total of 318 patients, older than 18 years of age, were included in the study. The patients were assigned into two groups according to age. The patients who were less than 65 years of age were assigned to Group I, and the patients who were 65 years or older were assigned to Group II. The laboratory values and ultrasonographic findings of the patients in these two groups were compared.

The results are expressed as the mean ± the standard deviation (SD). Comparisons of the data were performed using the Chi-square test and Fisher's exact test. The statistical analyses were performed using a software package SPSS Version 11.5 (SPSS Inc. Chicago). All P values less than 0.05 were considered statistically significant.

## 3. Results

Group I consisted of 225 patients, 132 females with a mean age of 45.18±13.27 years (range 20-62) and 93 males with a mean age of 44.58±12.07 years (range18-64). Group II consisted of 93 patients, 48 females with a mean age of 75.81±6.52 (range 67-88) and 45 males with a mean age of 77.35±8.41 years (range 66-91). The female to male ratio of the patients who were admitted with the complaint of right upper quadrant pain was 132 to 93 (1.42) in Group I and 48 to 45 (1.06) in Group II. The gender difference between the two groups was not significant. The diagnosis of acute cholecystitis was established in 39 of the 225 patients in Group I and in 36 of the 93 patients in Group II. There was a statistically significant difference between the two groups with respect to the final diagnosis (p<0.001) (Table 1).

The female to male ratio of the patients who were diagnosed to have acute cholecystitis was 27 to 12 (2.25) in Group I and 15 to 21 (0.71) in Group II. The gender difference between these two groups was also significant (p=0.016). All patients who were diagnosed as acute cholecystitis had gallstones without any difference between the young and elderly ones) (Table 2).

Ultrasonography showed gallstones in 117 of the 318 patients who were admitted with the complaint of right upper quadrant pain (Table 3). A total of 21 patients had single stone and 96 patients had multiple millimetric calculi or sludge. There was no significant gender and age difference regarding the type of gallstones in patients with diagnosis of acute cholecystitis.

There was no significant difference regarding body temperature between Group I and Group II with, respectively, 37.8°C (±0.2) and 37.5°C (±0.3).

**Figure 1:**
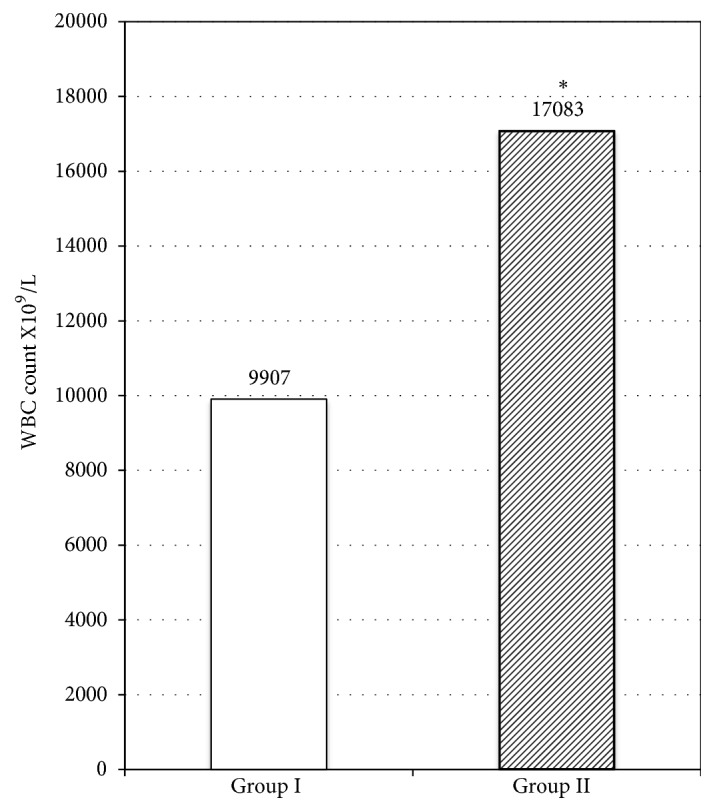
The average white blood cell count (WBC) of the patients with diagnosis of acute cholecystitis. (*∗*) The average white blood cell count (WBC) of the patients who were diagnosed to have acute cholecystitis was significantly higher in the elderly patient group (p<0.001).

Leukocytosis was present in 12 of 39 patients in Group I with acute cholecystitis and in 33 of 36 patients in Group II with acute cholecystitis (Table 4). The average white blood cell (WBC) count of the patients who were diagnosed to have acute cholecystitis was 9907±4.437 x 10^9^/L in Group I when it was 17083±7485.3 x 10^9^ /L in Group II. A significant difference was noted in the presence of leukocytosis when the age groups of the patients with acute cholecystitis were compared (p<0.001) (Figure 1). Serum biochemistry showed elevated bilirubin levels in 18 of 39 patients with acute cholecystitis in Group I and in 24 of 36 patients in Group II. Hepatic transaminases were also elevated in 15 of 39 patients with acute cholecystitis in Group I and in 18 of 36 patients with acute cholecystitis in Group II. There were no notable differences between Group I and Group II with respect to serum biochemistry.

When the laboratory findings of all patients with acute cholecystitis were analyzed it was observed that white blood cell (WBC) counts were elevated in 9 of 9 patients in the group of patients having a single stone and 36 of 66 patients in the group of patients having multiple calculi or sludge. The percentage of patients with elevated WBC count was significantly higher in the group of patients having a single gallbladder stone versus the group of patients having multıple calculi or sludge (p=0.009). On the other hand the percentages of the patients with elevated serum levels of hepatic transaminases and bilirubin were similar between these two groups of patients with respect to the type of gallstones.

## 4. Discussion

Abdominal pain is a common cause of emergency department (ED) admission in elderly patients. The evaluation of abdominal pain in the geriatric patients may be confounded by atypical presentation and physical examination, limitations in history taking, unreliable vital signs, and laboratory values [13]. The ability of physicians to determine the cause of abdominal pain decreases with advancing patient age. Acute cholecystitis is a very representative example of this scenario, as it is one of the most common causes of acute abdominal pain in the elderly [4, 5, 13].

In our study the gender difference between the young and elderly patient groups was significant in the diagnosis of acute cholecystitis. The female to male ratio is 2.25 in young group and 0.71 in elderly group (p=0.016). The diagnosis rate of acute cholecystitis in young female patients with RUQ pain is higher than in young males, but this difference decreases with age. This age-related difference between women and men was confirmed in many studies [14]. GREPCO study found a female to male ratio of 2.9 between the ages of 30 to 39 years, but the ratio narrowed to 1.6 between the ages of 40 and 49 years and to 1.2 between the ages of 50 and 59 years [15]. Nikfarjam et al. also reported that male patients with acute cholecystitis tended to be older [16]. Rates of gallstones are two to three times higher among women than men and the risk for gallstone disease in women is primarily higher in the childbearing age [17, 18]. After the fifth decade the rates of new gallstone formation in men and women become essentially equal [19]. Estrogen increases biliary cholesterol secretion causing cholesterol supersaturation of bile. Therefore, gallstone formation is influenced by some risk factors such as female gender, pregnancy, estrogen therapy, and oral contraceptives [14]. Probably because of these factors women clearly have a higher incidence rate of acute cholecystitis than men at young age, but this difference disappeares with increasing age. We should also mention that more women with gallstone disease are treated with cholecystectomy at a young age because of acute cholecystitis [20]. Even some studies suggest that acute cholecystitis is a different disease in women when compared with men [21, 22].

There was no significant difference regarding body temperature between young and elderly patients in our study. When we review the literature, in a study evaluating young and elderly patients with sepsis, body temperature ≥ 38°C was more common in the young group compared with the elderly group (63% versus 60%, respectively); body temperature < 37.2°C was found in 23% of the elderly patients. They reported that there was not any significant difference between the two groups in this regard [23]. Compared to younger infected patients, elderly ones with bacteremia had fewer signs or symptoms and mostly can not develop a fever, but instead may present with hypothermia. That is why it would be prudent to treat any elderly with possible cholecystitis as having a significant infection [24, 25]. This can be also the reason why there was no significant difference between young and elderly patients body temperature in our findings.

Once we reviewed the laboratory values of the patients diagnosed to have acute cholecystitis, we observed that, comparing to the young group, there was a greater proportion of elderly patients with leucocytosis. On analysis of our experience we underline that elderly patients with acute cholecystitis are more likely to demonstrate high level of white blood cells. This data may possibly be the result of delayed hospital admission of elderly people according to many coexisting health and social problems such as lack of health insurance or lack of transport to health care units. Akasu et al. reported leucocytosis in patients with acute cholecystitis, but no significant difference between young and elderly groups while Borzellino et al. reported leucocytosis in elderly patients with diagnosis of an acute cholecystitis, mean value of 15.6x10^9^/L as more similar to our study [26, 27].

In the elderly population, total WBC decreases slightly by aging but as response to an acute infection, sepsis, trauma, or inflammation the number of WBC may increase dramatically. In the presence of leukocytosis more than 14000 more attention should be paid to gastrointestinal, urinary, and skin infections in elderly patients with sepsis [23]. Some studies suggest that the predictive ability of theWBC counts also applies to the elderly but there is a need for further studies on this topic [28, 29].

We revealed that single gallbladder stones cause significantly higher WBC count than multiple calculi or sludge in elderly patients with acute cholecystitis (p=0.009). Previous studies mention that gallstones from most patients contain live bacteria with the potential to cause infective complications and solitary gallstones were found to develop after a precursor phase of over 2 years in contrast, multiple gallstones formed without a precursor phase [30, 31]. This could be why the development of mucocele, empyema, and perforation was significantly more common in patients with solitary gallbladder stones as Mofti et al. reported. Thus a patient with a solitary stone may need more attention and surgical priority [32].

The presentation of an older patient with acute cholecystitis may be very different from the presentation of the illness in a younger patient. The expected clinical picture may not be encountered in elderly patients. An incomplete or ambiguous history often complicates the assessment of existing symptoms. This is usually due to cognitive, functional, and sensory impairment seen in old patients. Diagnosing acute cholecystitis in geriatric patients having right upper quadrant pain can be misleadingly uncertain since they are likely to have coexistent disease with altered metabolic and endocrine responses [33]. In this point of view transabdominal ultrasonography was shown to be very accurate in diagnosing acute cholecystitis [11].

## 5. Conculusions

Elderly patients presenting with right upper quadrant abdominal pain are more likely to be diagnosed to have acute cholecystitis and they are more close to septic complications. We hope that our study will be convincing for clinicians to maintain a degree of awareness in the evaluation of geriatric patients with right upper quadrant abdominal pain.

## Figures and Tables

**Table 1:** The young (Group I) and elderly (Group II) of patients who were admitted with the complaint of right upper quadrant pain.

	Group I	Group II	
Patients with Right Upper Quadrant Pain	225	93	
Female	132	48	
Male	93	45	
Female/Male Ratio	1.42	1.06	
Presence of stone	69	48	
Single stone	9	12	
Multiple calculi or sludge	60	36	
Diagnosis of Acute Cholecystitis	39	36	p<0.001*∗*
Other Reasons of Right Upper Quadrant Pain	186	57	

(*∗*) There was a significant difference between the young and elderly patient groups with respect to the final diagnosis of acute cholecystitis in the presence of right upper quadrant pain (p<0.001).

**Table 2:** Patients with diagnosis of acute cholecystitis in young (Group I) and elderly (Group II) patients with right upper quadrant pain.

	Group I	Group II	
Patients with Right Upper Quadrant Pain	225	93	
Diagnosis of acute cholecystitis	39	36	p<0.001 *∗*
Presence of stone	39	36	
Female	27	15	
Male	12	21	
Female/Male (Ratio)	2.25	0.71	P=0.016 *∗∗*

(*∗*) There was a significant difference between the young and elderly patient groups with respect to the final diagnosis of acute cholecystitis in the presence of right upper quadrant pain (p<0.001).

(*∗∗*) The gender difference between the young and elderly patient groups was significant in the diagnosis of acute cholecystitis (p=0.016).

**Table 3:** Presence of gallbladder stones in patients who were admitted with the complaint of right upper quadrant pain.

	Young Group	Elderly Group	Total
Single stone	9	12	21

Multiple calculi or sludge	60	36	96

Total	69	48	*117*

Ultrasonography showed gallstones in 117 of the 318 patients (36.79%) who were admitted with the complaint of right upper quadrant pain. A total of 21 (17.94%) patients had single stone and 96 (82.05%) patients had multiple millimetric calculi or sludge in both young and elderly groups.

**Table 4:** Leukocytosis, bilirubin, and transaminase level in the young and elderly group of patients with a diagnosis of acute cholecystitis.

	Group I	Group II	
Number of patients	*39*	*36*	
Leukocytosis	12	33	p<0.001 *∗*
Elevated bilirubin level	18	24	
Elevated hepatic transaminases level	15	18	

(*∗*) A significant difference was noted in the presence of leukocytosis when the age groups of the patients with acute cholecystitis were compared (p<0.001).

## Data Availability

The data used to support the findings of this study are restricted by the Ethics Committee of the University of Ankara in order to protect patient privacy. Data are available from Müge Günalp (gunalpmuge@yahoo.com) for researchers who meet the criteria for access to confidential data
